# Within-Population Genetic Structure in Beech (*Fagus sylvatica* L.) Stands Characterized by Different Disturbance Histories: Does Forest Management Simplify Population Substructure?

**DOI:** 10.1371/journal.pone.0073391

**Published:** 2013-09-05

**Authors:** Andrea Piotti, Stefano Leonardi, Myriam Heuertz, Joukje Buiteveld, Thomas Geburek, Sophie Gerber, Koen Kramer, Cristina Vettori, Giovanni Giuseppe Vendramin

**Affiliations:** 1 Plant Genetics Institute, National Research Council, Sesto Fiorentino, Italy; 2 Dipartimento di Bioscienze, Università di Parma, Parma, Italy; 3 Department of Forest Ecology and Genetics, CIFOR-INIA, Madrid Spain; 4 Alterra, Wageningen, The Netherlands; 5 Federal Research Centre for Forests, Vienna, Austria; 6 UMR 1202 BIOGECO, INRA, Cestas, France; 7 UMR 1202 BIOGECO, University of Bordeaux, Talence, France; University of Umeå, Sweden

## Abstract

The fine-scale assessment of both spatially and non-spatially distributed genetic variation is crucial to preserve forest genetic resources through appropriate forest management. Cryptic within-population genetic structure may be more common than previously thought in forest tree populations, which has strong implications for the potential of forests to adapt to environmental change. The present study was aimed at comparing within-population genetic structure in European beech (*Fagus sylvatica* L.) plots experiencing different disturbance levels. Five plot pairs made up by disturbed and undisturbed plots having the same biogeographic history were sampled throughout Europe. Overall, 1298 individuals were analyzed using four highly polymorphic nuclear microsatellite markers (SSRs). Bayesian clustering within plots identified 3 to 11 genetic clusters (within-plot *θ*_ST_ ranged from 0.025 to 0.124). The proportion of within-population genetic variation due to genetic substructuring (*F*_CluPlot_ = 0.067) was higher than the differentiation among the 10 plots (*F*_PlotTot_ = 0.045). Focusing on the comparison between managed and unmanaged plots, disturbance mostly explains differences in the complexity of within-population genetic structure, determining a reduction of the number of genetic clusters present in a standardized area. Our results show that: *i*) genetic substructuring needs to be investigated when studying the within-population genetic structure in forest tree populations, and *ii*) indices describing subtle characteristics of the within-population genetic structure are good candidates for providing early signals of the consequences of forest management, and of disturbance events in general.

## Introduction

Within-population genetic structure is shaped by the complex interplay of genetic and demographic factors. In forest tree populations subject to anthropogenic influence, management can alter both processes influencing the amount and distribution of genetic variation [1]–[4]. Within-population genetic structure follows spatial as well as temporal dynamics whose comprehension is one of the bases for understanding how populations evolve [5]. In addition, it has recently been pointed out that the study of within-population genetic structure is a key prerequisite to correctly interpret the results of association genetic studies, since cryptic genetic structure can yield spurious statistical associations between genotypic and phenotypic traits [6], [7]. The probability of such misinterpretations is particularly high when studying forest tree populations because they have long been seen as large, random-mating units with minimal structure [8].

The partitioning of within-population genetic diversity into well-distinguished genetic clusters indicates the existence of subpopulations (i.e. ‘population stratification’, [9], [10]). However, genetic clusters are not necessarily spatially clumped, since individuals belonging to the same genetic cluster may be spatially randomly distributed, and clusters may be highly intermingled. In forest trees, the focus of most studies has mainly been directed towards the spatial component of within-population genetic structure, while its non-spatial component has only rarely been assessed [5]. Slavov et al. [7] surprisingly found a strong non-spatial genetic substructure in a small continuous *Populus trichocarpa* stand characterized by an extensive gene flow via pollen. The stand was composed of individuals that were unambiguously assigned to two distinct subpopulations that probably originated from different seedling cohorts. Jansson & Ingvarsson [8] underlined how such cryptic structures may be more common than previously thought in forest tree populations. Besides temporally separated founder events during colonization, other temporal factors, such as phenological and recruitment dynamics, can determine non-spatial clustering of genetic variation. In addition, re-afforestation using mixed seed lots from distinct provenances and other silvicultural practices might also result in non-spatial clustering of genotypes.

Natural and anthropogenic disturbances can alter the distribution of genetic variation within populations. This variation is essential for the potential of forests to adapt to environmental change, such as climate change [11]–[13]. The comprehension of the consequences of disturbances, such as fires and silvicultural practices, on within-population genetic structure can therefore be crucial to preserve forest genetic resources through appropriate management [4], [14]. A few studies investigated the effects of common forest management practices on the within-population genetic structure. In some cases, a negative impact of management practices on genetic diversity and spatial genetic structure (SGS hereafter) was shown [3], [15]–[18], although no effect or a weak effect on gene flow, genetic diversity and SGS was found elsewhere (e.g. [17], [19], [20]). The effects of some regeneration practices on genetic diversity was demonstrated to be species-specific [17] and, interestingly, even population-specific [15], [19].

The lack of experiments specifically designed to compare the within-population genetic structure between disturbed and undisturbed forest tree stands has retarded our understanding of the fine-scale consequences of forest management [21], [22]. The present study was aimed at comparing stands experiencing different disturbance levels using five plot pairs of *Fagus sylvatica* (L.). In each pair, plots were chosen as geographically close as possible to avoid the effect of potential confounding factors. An exhaustive spatial sampling allowed us to perform an in-depth analysis of within-population genetic structure using different approaches (spatial autocorrelation analysis and Bayesian clustering) that have proved effective for revealing cryptic genetic structuring. Therefore, the specific aims of the study were: *i*) to investigate the existence and, in case, the characteristics of within-population genetic structure in the ten plots analyzed, and *ii*) to check whether disturbance had an impact on within-population genetic structure in each plot pair.

## Materials and Methods

### Study Sites, Sample Collection and Microsatellite Analysis

Study sites and sampling strategy have been described in details in previous works [23]–[26]. Briefly, five study sites were chosen in five European countries: Austria, France, Germany, Italy and The Netherlands. Each study site consists of 2 plots located in close proximity of each other, with the exception of the French ones which are also characterized by a marked difference in altitude (Table 1, Table S1). In the other 4 sites, environmental conditions, climate and genetic background are assumed to be similar between plots. Within each site, the 2 plots differ in the intensity of natural or anthropogenic disturbance, mainly due to forest management practices. Differences in stand structure were generally marked except between the Dutch plots (Table S1). The undisturbed Dutch plot has been subject to low-intensity human management during the last three centuries (cattle grazing, fire wood and construction wood for local use) with a gradual cessation of human activities. However, the managed Dutch plot is a plantation, probably established in 1870 with local forest reproductive material. Therefore, the recent management histories of the two plots are markedly different (Table 1).

**Table 1:** Characteristics of investigated beech plots.

Country	Plot	Code^a^	Disturbance history	N	Plot size(ha)	Density(trees/ha)	Latitude/Longitude	Altitude(m)	Standage
Germany	Flecken-Zechlin 1	Gl	Semi-natural	120	0.86	140	53°11/12°43′	85	75–140
	Flecken-Zechlin 2	Gh	Shelterwood	120	0.91	132	53°11/12°44′	85	46–155
The Netherlands	Pijpebrandje	NLl	Semi-natural	120	0.68	178	52°15′/5°43′	50	130–200
	Solse Bosje	Nlh	Plantation	120	0.91	132	52°14′/5°39′	50	130
Austria^b^	Dobra1	Al	Natural	110	1.87	58	48°35′/15°23′	390–550	250–300
	Dobra2	Ah	Shelterwood	110	0.58	191	48°35′/15°23′	550–580	–
France	St. Baume	Fl	Natural	286	1.91	150	43°19′/5°45′	750	–
	Mt. Ventoux	Fh	Colonisation	90	1.32	68	44°10′/5°16′	1450	–
Italy^c^	Abruzzo A	Il	Natural	112	0.56	196	42°30′/13°29′	1270	–
	Abruzzo C	Ih	Coppice before 1850,then shelterwood	110	0.21	537	42°30′/13°29′	1155	70

a Plot codes were formed by the indication of country (G = Germany, NL = The Netherlands, A = Austria, F = France, I = Italy) and intensity of disturbance (l = low, h = high).

b Austrian plots are subplots of Piotti et al. [26] plots.

c Italian plots studied by Paffetti et al. [25] are subplots of the ones analyzed here.

All 1298 sampled individuals were genotyped at four highly polymorphic unlinked SSRs (FS1–15, FS4–46, FS1–25 and mfc5, [24]). The four SSRs displayed a total of 25, 41, 26, 31 alleles, respectively, and provided exclusion probabilities as high as ∼0.985 when used for paternity analysis in French plots [26]. Non negligible frequencies of null alleles have been reported previously for some marker-site combinations [26], [27].

### Data Analysis

General estimates of genetic diversity as well as genetic differentiation and phylogeographic analyses are reported in Buiteveld et al. [24]. We recalculated inbreeding coefficients (*F*_IS_) taking into account the possible presence of null alleles by using the program INEst, running the individual inbreeding model with a Gibbs sampler of 10^5^ iterations [28].

Fine-scale within-population genetic structure was investigated following two approaches: classical spatial autocorrelation analysis and Bayesian clustering.

Spatial autocorrelation analysis was performed using the multivariate method by Smouse & Peakall [29] implemented in the program GenAlEx 6.5 [30]. It provides a multi-locus estimate of pair-wise relatedness between individuals (*r*) which minimizes the stochasticity found in single locus or single allele estimates of relatedness. Tests for statistical significance were conducted by *i*) random shuffling (1000 times) of individual geographic locations to define the upper and lower bounds of the 95% confidence interval for each distance class, and *ii*) estimating 95% confidence intervals around mean *r* values by bootstrapping pair-wise comparisons within each distance class (1000 repeats). Analyses were performed using both the even distance classes option (using 20 m wide distance classes) and the even sample size option (distributing all possible pairs in seven distance classes with similar numbers of pairs per class). The nonparametric heterogeneity test proposed by Smouse et al. [21] was used to compare correlograms between plots within each pair, setting the number of bootstrap resamplings to 9999.

The intensity of SGS was also measured by the *Sp* statistic [31]. *Sp* is computed as *Sp* = *b*_F_/(*F*_1_–1), where *b*_F_ is the regression slope of the kinship estimator *F*_ij_ computed among all pairs of individuals against their geographical distances, and *F*_1_ is the average kinship coefficient between individuals of the first distance class (0–20 m). *Sp* has the desirable characteristic of being comparable among stands in a single study and among studies. The statistical significance of *F*_1_ and *b*_F_ was tested based on 1000 permutations of individual locations among individuals. All analyses were performed using SPAGeDi 1.3 [32].

To assess the power of the marker set to detect SGS in the studied stands we performed spatially explicit simulations of a population life cycle (pollen and seed dispersal, reproduction, death and replacement) for 64 generations following the simulation approach described in Heuertz et al. [33] and De-Lucas et al. [34]. At generation null, individuals were given random genotypes at microsatellite loci according to the observed allele frequencies in stand Fh (the plot with the strongest SGS). Spatial genetic structure was then allowed to build up over generations according to four scenarios characterized by different combinations of dispersal parameters (the axial standard deviation of gene dispersal, σ_g_) and density, spanning from an unrealistically high SGS for beech (*F*_1_>0.09) to very low SGS (*F*_1_<0.01). Intermediate scenarios were chosen to mimic SGS in the 2 French plots, according to pollen flow parameters and effective number of pollen donors estimated in the same plots [26] and seed dispersal parameters from Oddou-Muratorio et al. [35] and Millerón et al. [36]. For the low-SGS scenario, a parameter combination that produced an almost absent SGS was selected. On the other hand, for the high-SGS scenario, dispersal and density values significantly higher than the highest found among the study plots were chosen. In the simulation procedure the generation overlap was 50%. Sixty-four generations were modelled, taking measures of SGS at generations 1, 2, 4, 8, 16, 32, 48, and 64 and using a sampling scheme similar to the Fh stand. Simulations were repeated 100 times with 4 and 20 loci (5 independent replicates of the original 4 loci).

The simulation results were used to assess possible errors introduced by the low number of loci by: *i*) Comparing the no-structure confidence intervals observed in Fh and Fl with the distribution of simulated *F*_1_ values from intermediate scenarios. The rationale is that the four microsatellites have sufficient power to detect SGS if SGS estimates from realistic simulations fall consistently outside the no-SGS 95% confidence interval. *ii*) Comparing *F*_1_ values observed in Fh and Fl with those obtained from the simulated extreme scenarios. The overlap of *F*_1_ values from the observed *vs.* simulated scenarios will inform on the power of the four loci to discriminate between realistic and unrealistic SGS scenarios. *iii*) Comparing simulations with four and 20 loci. This comparison will help us assess the gain in accuracy and precision of SGS estimates when increasing the number of SSR loci.

The spatially explicit Bayesian clustering algorithm implemented in the R package GENELAND v. 4.0.2 was used to assign individuals to putative sub-populations within each plot [37]. We used the spatial model with correlated allele frequencies, setting the maximum number of sub-populations to 20, and running the analyses for 10^6^ iterations, with a thinning value of 1000. The possible presence of null alleles was explicitly taken into account by using the filter.null.alleles option. Each analysis was repeated 10 times. The highest median number of clusters of the 10 repetitions was chosen as the most representative one for each plot (see also Figure S1). To test whether clusters identified by Bayesian clustering were differentiated, Weir & Cockerham [38] *θ*_ST_ among clusters within each plot was calculated with Fstat [39]. Statistical significance of *θ*_ST_ values was tested after 1000 randomization of genotypes among clusters. To account for the possible presence of null alleles, differentiation among clusters was also calculated with FreeNA applying the ENA correction method to correct efficiently for the positive bias induced by the presence of null alleles on *θ*_ST_ estimation [40].

Once reliable Bayesian clustering results were obtained, a hierarchical analysis of the genetic structure of all plots was performed to assess the importance of population substructure relative to structure at higher hierarchical scales. The hierarchical estimates of *F*-statistics were obtained by the R package *HIERFSTAT* [41] using the method by Weir & Cockerham [38] based on the estimation of variance components of gene frequencies. Two intermediate hierarchical levels were chosen: plot (Plot) and clusters (Clu). Therefore, in the following *F*_PlotTotal_ refers to the correlation of genes within plots relative to the total, *F*_CluPlot_ to the correlation of genes within clusters relative to the plots, and *F*_IndClu_ to the correlation of genes within individuals relative to the clusters. *F*-statistics are related to each other by the following expression (1−*F*_IndTotal_) = (1−*F*_IndClu_)×(1−*F*_CluPlot_)×(1−*F*_PlotTotal_). Statistical significance of *F*-statistics was tested using 1000 randomization of the units defined by the level just below that of interest in the hierarchy.

Plots with larger areas are expected to contain a higher number of clusters just by chance. To compare the number of clusters produced by GENELAND among sites with different areas, we calculated 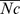, that is the mean number of clusters (*Nc*) in a standardized area of 0.21 ha (equal to the area of Ih, the smallest of our plots) in each plot. Since there is very large number of ways to subset an area, we performed the standardization procedure as follows: in each plot, the *Nc* was recorded within a window of 0.21 ha drawn around each individual and the number of clusters was then averaged for all windows around all individuals. To avoid border effects, *Nc* was calculated only for individuals more than 23 m from the border (<*i.e.* half-side of a 0.21 ha window). 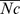 and its confidence interval was then calculated for each plot as a measure of complexity of the genetic structure.

## Results

**Figure 1:**
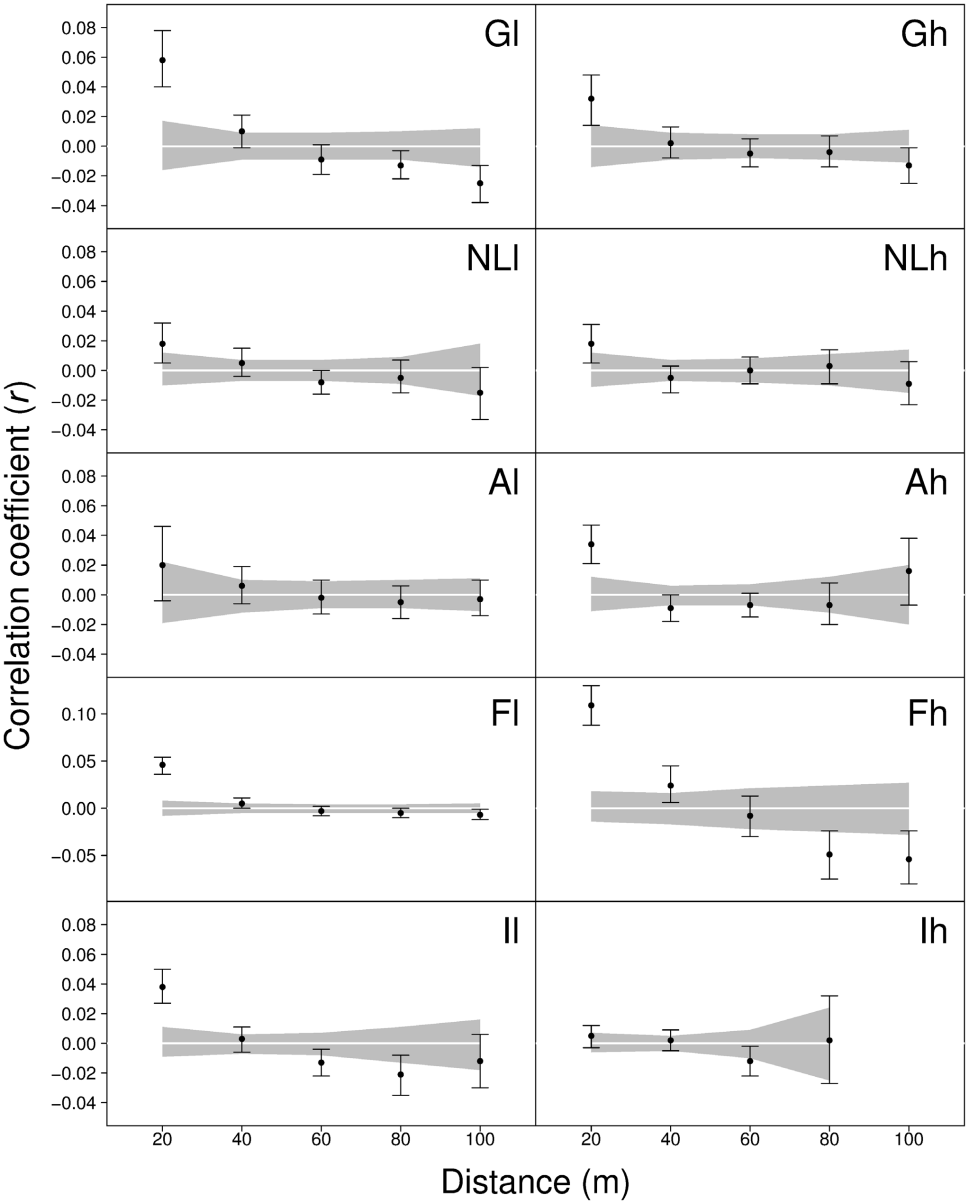
Correlograms from spatial autocorrelation analysis using the correlation coefficient *r* by Smouse & Peakall [29] and even distance classes. Shaded areas represent the 95% confidence interval obtained through random shuffling (1000 times) of individual geographic locations, black lines around mear *r* values represent 95% confidence intervals around mean r values generated by bootstrapping (1000 times) pair-wise comparisons within each distance class.

**Figure 2:**
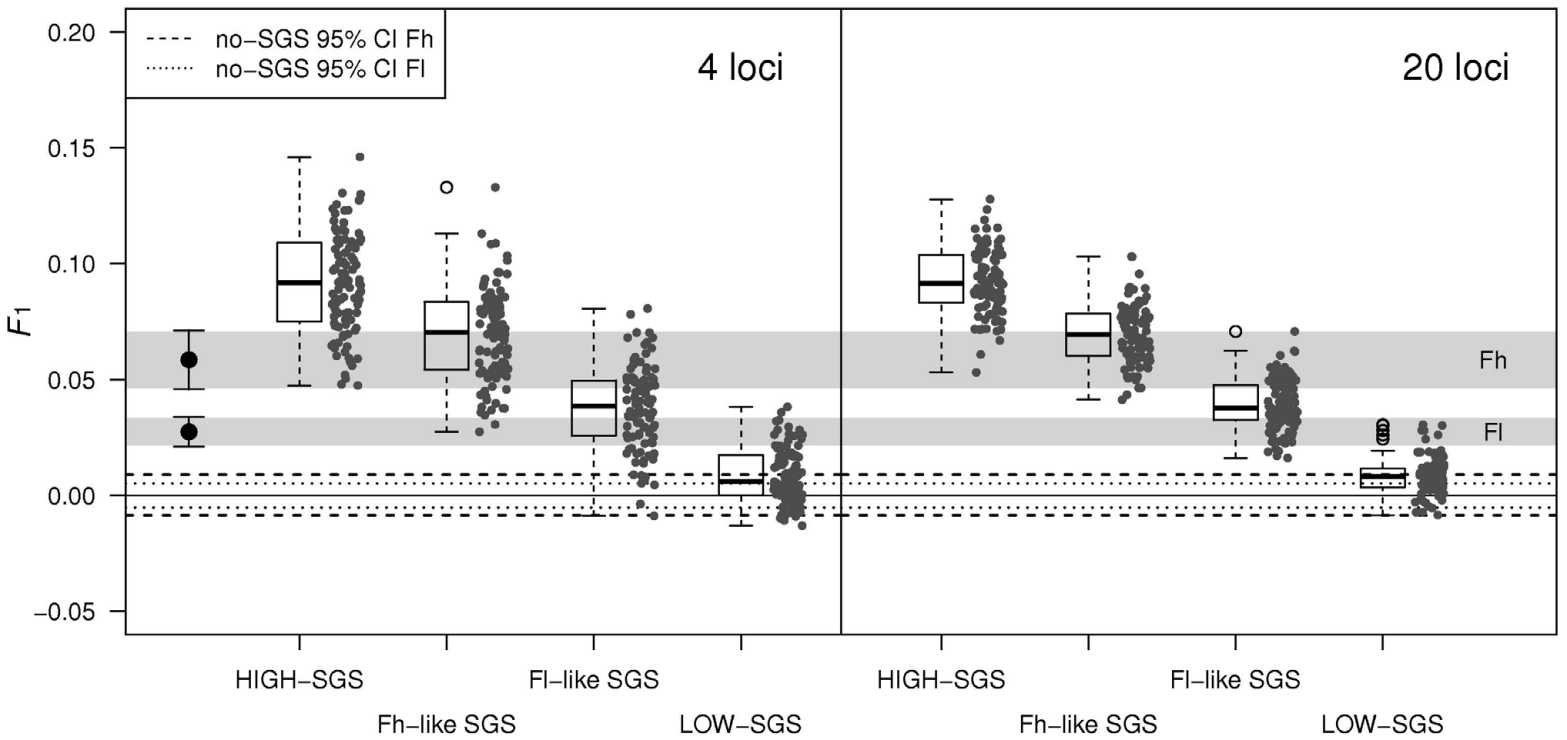
Assessment of the power of the marker set to detect SGS by spatially explicit simulations. For illustration of the results, the distribution of the kinship coefficient *F*_1_ between neighbours at generation 64 was used as the focal statistic (grey dots and boxplots) and compared to *i*) the no-structure 95% confidence intervals of *F*_1_ from the Fh and Fl populations (dotted lines, see legend in the left panel) obtained by random shuffling of individual geographic locations, and *ii*) real *F*_1_ values from Fh and Fl (black dots in the left panel) and their confidence intervals (grey areas). Results from simulations with 4 and 20 loci (right and left panels, respectively) are reported. Parameter settings for the 4 simulated scenarios were σ_g = _12 m and D = 20 trees/ha (HIGH-SGS), σ_g = _12 m and D = 35 trees/ha (Fl-like SGS), σ_g = _29 m and D = 50 trees/ha (Fh-like SGS), σ_g = _72 m and D = 145 trees/ha (LOW-SGS).

Spatial autocorrelation analyses based on different methods to estimate pair-wise genetic relatedness were concordant in detecting a significant spatial genetic structure in the first distance class (0–20 m) in all plots with the exception of Al and Ih, where no evidence of SGS was observed (Figure 1). Correlograms were similar using the ‘even sample size’ or the ‘even distance class’ option (results not shown). Fh is the only plot where SGS extends up to 40 m. Simulations confirmed the robustness of results from spatial correlation analysis with respect to the power of the marker set used (Figure 2) and the presence of null alleles (Text S1). In particular, Figure 2 shows that: *i*) the *F*_1_ values computed from the spatially explicit simulations for scenarios with intermediate strength of SGS fell consistently outside the no-SGS 95% confidence intervals obtained by random shuffling of individual geographic locations, *ii*) the confidence intervals of observed *F*_1_ values in Fh and Fl showed a limited overlap with *F*_1_ values from simulated extreme scenarios, with an error rate not higher than 19%, and *iii*) increasing the number of loci from 4 to 20 moderately increased the precision but had no effect on accuracy: mean values did not significantly differ between 4 and 20 loci scenarios as assessed by t-tests with Welch modification for unequal variances between groups (HIGH-SGS scenario: *t = *0.38, df = 168.7, *P = *0.70; Fh-like SGS scenario: *t = *0.25, df = 167.0, *P = *0.80; Fl-like SGS scenario: *t = *0.75, df = 163.9, *P = *0.46; LOW-SGS scenario: *t* = −0.20, df = 171.3, *P = *0.84). Therefore, we inferred that the 4 microsatellite loci have high power to detect SGS in a dispersal context that is realistic for the beech stands studied in this work.

The highest *F*_1_ values were recorded for Fh, Gl and Fl, the last two being the only plots with a statistically significant excess of homozygotes (Table 2). The *Sp* statistic ranged from 0.0040 in Ih to 0.0293 in Fh and, in general, it reflected results from spatial autocorrelation analyses obtained from GenAlEx: *Sp* was high in plots where genetic variability is spatially structured (Gl, Fh, and Il) whereas the lowest *Sp* values were recorded in plots characterized by an absence of SGS (Al and Ih). In pair-wise comparisons, correlograms of the Fh-Fl and Ih-Il pairs were statistically different. This was mainly due to a large difference in the first distance class, with r_1,Fh_>r_1,Fl_ and r_1,Il_>r_1,Ih_ (Table S2, Figure 1).

**Table 2:** Parameters describing within-population genetic structure in the studied beech plots.

Site	SGS parameter			*Nc*	*θ*_ST_	*F*_IS_
	*F*_1_	*b*_F_ (±SE)	*Sp* (±SE)			
Gl	0.0371***	−0.0258±0.0049***	0.0268±0.0043	9	0.087***	0.170±0.055***
Gh	0.0187***	−0.0115±0.0048***	0.0117±0.0076	7	0.025***	0.025±0.025
NLl	0.0115***	−0.0101±0.0021***	0.0102±0.0041	10	0.105***	0.068±0.055
NLh	0.0104**	−0.0095±0.0031***	0.0096±0.0052	3	0.062***	0.063±0.042
Al	0.0111	−0.0068±0.0015**	0.0069±0.0059	8	0.043***	0.051±0.040
Ah	0.0183***	−0.0131±0.0032***	0.0133±0.0034	8	0.042***	0.023±0.022
Fl	0.0274***	−0.0096±0.0017***	0.0099±0.0033	11	0.050***	0.083±0.038*
Fh	0.0585***	−0.0276±0.0025***	0.0293±0.0065	9	0.124***	0.033±0.028
Il	0.0224***	−0.0186±0.0039***	0.0190±0.0049	8	0.049***	0.042±0.036
Ih	0.0015	−0.0040±0.0035*	0.0040±0.0024	3	0.043***	0.023±0.022

*F*_1_, average kinship coefficient between individuals of the first distance class (0–20 m); *b*_F_, regression slope of the kinship estimator *F*_ij_ computed among all pairs of individuals against geographical distances; *Sp*, intensity of SGS; *Nc*, mean number of clusters from GENELAND analyses*; θ*_ST_, differentiation among clusters within each plot; *F*_IS_, inbreeding coefficient estimated by INEst.

* *P*<0.05,

** *P*<0.01,

*** *P*<0.001.

The highest median number of clusters into which individuals are grouped by Bayesian clustering (averaged over 10 independent GENELAND runs) ranged from 3 (Ih and NLh) to 11 (Fl) (Table 2, Figure S1). Within-plot *θ*_ST_ among GENELAND clusters ranged from 0.025 (Gh) to 0.124 in the recently colonized plot (Fh), with several plots showing high within-plot differentiation (Tab. 2). The *θ*_ST_ values estimated applying the ENA correction method using FreeNA were not different from the ones without correction (Paired *t*-test, *t = *0.44, df = 9, *P = *0.67), indicating a negligible influence of the possible presence of null alleles on differentiation estimates. Differentiation among clusters within plots (*F*_CluPlot_ = 0.067, *P*<0.001) was actually larger than the differentiation among plots, which is determined by both post-glacial recolonization history at the biogeographical scale and disturbance history at the local scale (*F*_PlotTot_ = 0.045, *P*<0.001). *F*_IndClu_ was 0.186, including the effect of both inbreeding (*F*_IS_>0) within clusters and null alleles.

**Figure 3:**
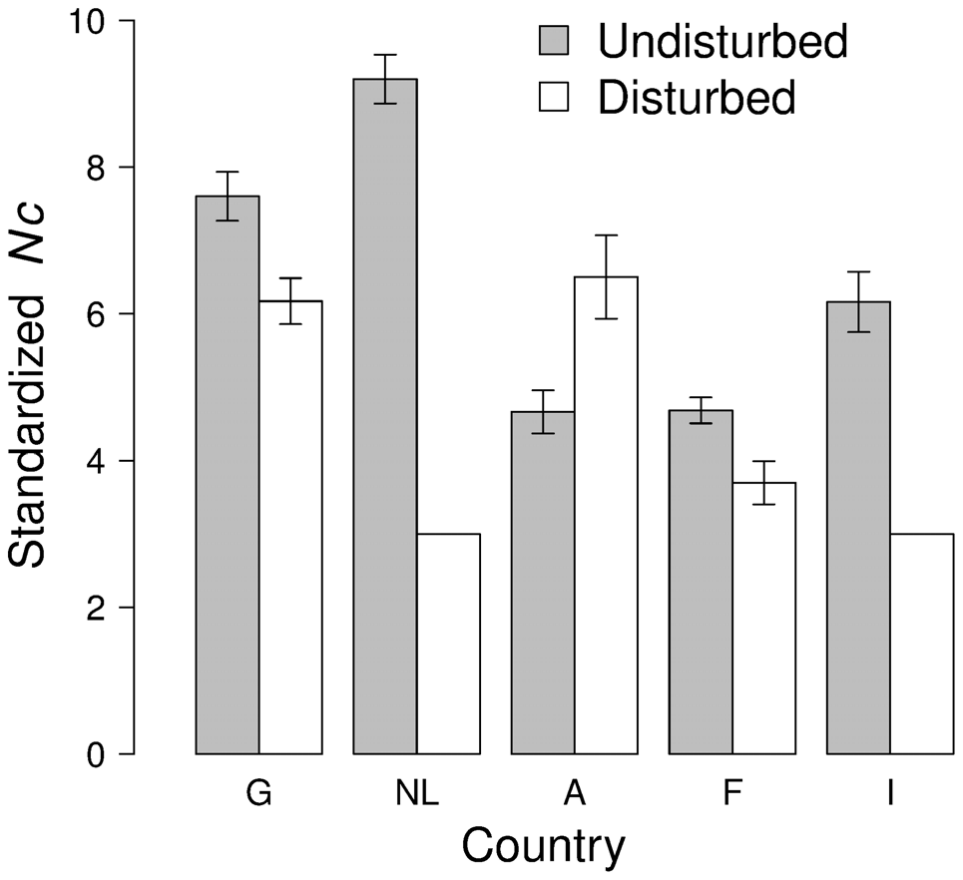
Complexity of within-population genetic structure as measured by the standardized number of clusters in an area of 0.21 ha. 0.21

Standardizing the number of clusters over an area of 0.21 ha (the area of the smallest plot, Ih), we found that undisturbed plots are generally characterized by a more complex structure (Figure 3). The mean 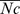 was 9.20 and 6.16 in Dutch and Italian undisturbed plots (NLl and Il), respectively, about three and two times the mean 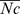 observed in nearby disturbed plots (3.00). Mean 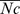 was higher in the disturbed compared to the undisturbed plot only in Austria (6.50 and 4.66, respectively).

## Discussion

Our experimental setup allowed us to obtain a reliable set of parameters describing the within-population genetic structure of European beech in a wide variety of ecological and management conditions, spanning from undisturbed to highly managed stands. Two relevant results for the comprehension of within-population dynamics in *F. sylvatica* were achieved: *i*) despite the variety of conditions explored, we found that a large proportion of within-population genetic variation is due to genetic substructuring in all plots, and *ii*) in plot pairs sharing the same bio-geographic history, disturbance explains most of the difference in the complexity of within-population genetic structure.

### Within-population Genetic Structure in Beech

Our work confirms what emerged in previous studies, that SGS in beech extends up to 40 m and not further [35], [42]–[44]. An SGS up to larger distances was found only with AFLPs [45]. This is likely to be related to the peculiar characteristics of these markers, namely the much higher number of loci that can be scored. In fact, Jump et al. [44] concluded that SGS estimates should only be compared within marker types and for similar marker numbers. According to the theory of isolation by distance, the intensity of SGS is negatively related to effective density (*d_e_*) and dispersal distances [31]. European beech has a high potential for pollen dispersal, with large (∼75%) pollen immigration within small to medium size (1–8 ha) plots and mean within-population pollen dispersal distances ranging from 40 to 180 m in natural stands [5], [26], [35]. Seed dispersal is much more limited. In general, seed immigration from outside the plot is <30% and mean seed dispersal distance is approximately 10 m [5], [35]. Chybicki et al. [43] estimated a ratio of seed to pollen dispersal distances ranging from 1∶10 to 1∶100 for beech. Our results showed how the interplay between limited seed dispersal and large pollen flow is likely to have determined the spatially restricted SGS (up to 20 m) detected in all plots at regular tree density (100<*d*<200 trees ha^−1^). The lack of a spatial signal in the genetic structure of Ih could have been a result of high local density, which should result in a considerable overlap of individual gene shadows. Conversely, the SGS up to 40 m in Fh, an area characterized by high pollen immigration (∼80%, [26]), can be explained by the low density in this colonization area. This is in agreement with what was previously found in beech colonization areas [35]. High SGS in Fh could also be an effect of local pollen dispersal distances being extremely low [26], further reducing the overlap between individual gene shadows. In Al, a low density plot from a pristine part of the Dobra forest, we curiously found an absence of SGS. This result exemplifies that an observed SGS pattern can result from a series of determinants that affect spatial patterns [1], [2], and how their interaction can determine unexpected results. For instance, Vekemans & Hardy [31] noted in several studies a positive, and thus counterintuitive, effect of density on the intensity of SGS. On the other hand, dispersal can be strongly enhanced when density is low and, as a consequence, its effect can prevail over the density effect *per se* in shaping SGS.

Our results together with previous studies [35], [43]–[44] allowed us to obtain a reliable distribution of *Sp* values (*n* = 19), ranging from 0.0054 in a low intensity managed stand [44] to 0.0354 in an expanding population [35]. About 60% of studied stands are characterized by *Sp* values between 0.005 and 0.015, confirming the general conclusion by Chybicki et al. [43] that *Sp* in *F. sylvatica* is around 0.01, which is typical for outcrossing species whose pollen is wind-dispersed. Still, according to Vekemans & Hardy [31], values of *Sp* between ∼0.01 and ∼0.035 characterize species whose seeds are dispersed by wind and gravity, respectively. The distribution of *Sp* values suggests that seed dispersal might not be consistently limited in beech. In fact, although within-population seed dispersal distances are usually limited to a few dozens of meters, long distance dispersal can be strongly enhanced by secondary dispersal by birds, reaching up to several kilometres in beech [46], [47]. In an expanding oak (*Quercus robur*) population, for instance, seedlings growing under their mothers were responsible for high kinship at short distance whereas dispersed seeds accounted for most of the SGS pattern at distances beyond tens of meters [48]. In addition, although *F*_1_ is likely to be mainly determined by seed dispersal, low *Sp* values can also, in part, be determined by the capability of long distance pollen dispersal in beech.

A relevant result emerging from our survey is that local substructure explained a larger proportion of genetic variation (*F*_CluPlot_ = 0.067 and 0.025<*θ*_ST_<0.124, Table 2) than differentiation among all ten plots (*F*_PlotTot_ = 0.045). Notably, a relevant local substructure is present when the spatial signal is weak or even absent, making spatial autocorrelation analysis insufficient to characterize all aspects of population stratification at the local scale. Contrarily to what was previously thought, the presence of a complex within-population structure is emerging as a common characteristic in forest trees, even though until now few studies have addressed this topic and contrasting results have been found [7], [25], [49]. For instance, a single genetic cluster was found when analyzing a large low-density *P. trichocarpa* population whereas, in the same study, a small population surprisingly showed a clear substructure [7].

The presence of neutral genetic structuring at any geographical level can have profound implications for experimental design in studies aimed at searching phenotype–genotype associations and molecular signatures of selection [6], [50]. In fact, even a subtle neutral genetic differentiation observed at the population spatial scale needs to be statistically accounted for in association studies to avoid spurious associations [51]. Once stratification has been demonstrated, refined methods such as mixed models are now available to take into account even the hierarchically lowest stratification (i.e. family structure) in association studies [6].

### Effect of Disturbance on the within-population Genetic Structure

Our results indicate that disturbance histories had a site-specific influence on within-population genetic structure at a finer level than what is generally detected by methods commonly employed for investigating genetic consequences of forest management. In the literature, several studies aimed at comparing genetic diversity, SGS and gene flow between managed and unmanaged stands, or among stands managed by different techniques, are present (e.g. [3], [16], [20], [24]). Some contrasting results have been found but, in general, effects of silvicultural practices on the adult cohort are weak or absent (e.g. [24]). In the managed *vs.* unmanaged comparisons Gh-Gl, NLh-NLl, and Ih-Il, we detected a substantial reduction in the complexity of the genetic structure as measured by the mean number of clusters in a standardized area (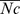) and by within-population *θ*_ST_ in disturbed plots. The reduction was low between the German plots, reflecting the recent divergence in their management history (Gl is a strict reserve only since 1961, [23]). The difference in the complexity of genetic structure was large in the comparison of the even-aged Dutch plantation (NLh) and the Italian formerly coppiced stand (Ih) with their respective unmanaged stands, indicating a possible high impact of such management regimes on the heterogeneity of within-population genetic structure in beech. In plantations, such a reduction can be generated by the use of a limited number of seed sources or genetically homogeneous material after a clear-cut [52], [53], which can also explain the weak SGS found by spatial autocorrelation analysis in NLh. In the Italian site, Ih has been subject to a complex recent management history: it was coppiced until 1850, then a conversion to high forest began through thinning from below and regeneration felling following the uniform shelterwood system [23], [25]. Such high anthropogenic pressure has produced the loss of the spatial component and a simplification of the non-spatial component of genetic structure in this stand, as also reported analyzing a subset of our Italian datasets with different methods and markers [25]. It is noteworthy, however, that this difference in the genetic structures does not correspond to a comparable reduction of within-population genetic diversity [24]. Paffetti et al. [25] reached similar results and the only difference in genetic diversity detected between managed and unmanaged stands was a slight reduction in the frequency of rare alleles in the managed stand.

The shelterwood system produced weak to negligible genetic effects in the stand pairs analyzed (Al-Ah, Gl-Gh), with no difference in genetic diversity [24] and only a slight reduction in the intensity of genetic structure in the German plot pair. Given the wide application of shelterwood-based techniques, the genetic consequences of this management system has been previously investigated and contrasting results have been found. Gene flow patterns were not affected by this silvicultural technique in *Pinus sylvestris* populations [20], whereas it decreased the SGS comparing three managed *P. strobus* stands with an old growth stand [3]. El-Kassaby et al. [17] found different genetic consequences of shelterwood in *Abies amabilis* and *Tsuga heterophylla*, two species with contrasting mating system dynamics. In two separate studies on *Pseudotsuga menziesii*, only one showed a reduction in genetic diversity in managed stands [15], [19]. This range of results shows that the response of forest trees to management techniques can be highly species-specific, and that several replicates of properly sampled plot pairs (managed *vs*. unmanaged) are needed to draw conclusions about the effects of natural and anthropogenic disturbances on the characteristics of within-population genetic structure.

Indices of genetic diversity as well as spatial structure and gene flow parameters are well suited for monitoring in long-term modelling studies, but detectable changes have rarely been found when comparing managed and unmanaged sites in beech [24], [26], [42]. Although not appropriate to describe changes in the evolutionary potential of populations, our results show that indices describing subtle changes in the within-population genetic structure (such as the mean number of clusters in a standardized area presented here) can be good candidates for providing early signals of the consequences of forest management on neutral genetic variation. A special effort should be put into properly characterizing within-population genetic structure in follow-up experimental and modelling studies for a deeper comprehension of short- and long-term responses of long-lived forest tree species to climatic as well as anthropogenic pressures.

## Supporting Information

Figure S1 **Bayesian clustering results.** Distribution of results from the 10 repetitions of GENELAND analyses for each plot. The highest median number of clusters of the 10 repetitions was chosen as the most representative one for each plot.
(TIF)

Table S1 **Stand characteristics of the 10 study plots.** For each social class the mean value of tree diameter at breast height and height, the stem number, the basal area and total volume are reported.
(PDF)

Table S2 **Heterogeneity tests of SGS between disturbed and undisturbed plots using GenAlEx (Peakall & Smouse** [**30**] **).** The reported values represent the degree of differentiation of SGS between two plots from the same site in each distance class (*t^2^*) and total *ω*.
(PDF)

Text S1 **Assessment of the potential impact of null alleles on spatial correlation analyses in the studied** ***Fagus sylvatica*** **stands.**
(PDF)
