# Supplementary material for: Within-Population Genetic Structure in Beech (Fagus sylvatica L.) Stands Characterized by Different Disturbance Histories: Does Forest Management Simplify Population Substructure?
Source: PLoS One. 2013 Sep 5;8(9):e73391. doi: 10.1371/journal.pone.0073391 (PMC3764177; doi:10.1371/journal.pone.0073391)
Supplement: Table S1 — Stand characteristics of the 10 study plots. For each social class the mean value of tree diameter at breast height and height, the stem number, the basal area and total volume are reported. (PDF) [file pone.0073391.s002.pdf]

Table S1. Stand characteristics of the 10 study plots. For each social class the mean value of tree diameter at breast height and height, the stem number, the basal area and total volume are reported.

Site	Social position[a]	Diameter [cm]		Height [m]		Stem number		Basal area [m^2]		Total volume [m^3]	
		Undisturbed	Disturbed	Undisturbed	Disturbed	Undisturbed	Disturbed	Undisturbed	Disturbed	Undisturbed	Disturbed
Austria	1	92.6	67.1	41.1	33.8	91	77	61.2	27.2	1680.4	582.5
	2	64.2	50.4	38.9	33.6	82	124	26.5	24.7	640.0	493.8
	3	65.2	39.3	39.8	33.2	22	34	7.4	4.1	190.7	77.3
	Total	78.6	55.1	40.4	33.7	201	235	97.5	56.1	2571.1	1153.6
Germany	1	64.8	63.2	31.6	32.1	25	22	8.2	6.9	162.5	138.5
	2	48.7	49.6	30.5	29.2	81	36	15.1	7.0	275.7	122.5
	3	35.4	20.6	28.5	22.3	44	132	4.3	4.4	71.3	58.4
	4	25.7	8.6	25.8	11.8	35	270	1.8	1.6	26.2	12.4
	5	8.7	4.6	15.0	8.6	112	508	0.7	0.8	6.1	5.5
	Total	36.6	16.4	30.0	26.5	316	981	33.3	20.8	602.4	338.5
France	1	57.7	34.1	27.9	19.8	34	20	8.9	1.8	155.1	23.3
	2	45.4	24.5	26.1	16.5	80	15	12.9	0.7	204.5	7.2
	3	33.8	20.1	23.1	15.1	18	6	1.6	0.2	22.2	1.8
	Total	47.6	29.1	26.6	18.6	132	41	23.5	2.7	381.8	32.3
Italy	1	100.4	42.0	33.1	26.7	32	1	25.3	0.1	586.4	2.2
	2	53.5	31.2	30.4	25.0	46	89	10.3	6.8	193.3	98.8
	3	30.0	20.1	25.4	20.9	8	25	0.6	0.8	8.2	9.5
	4	17.3	17.7	19.4	19.5	25	19	0.6	0.5	6.7	5.2
	5	10.7	-	11.4	-	7	-	0.1	-	0.5	-
	Total	63.1	28.0	32.0	24.3	118	134	36.9	8.2	795.2	115.7
The Netherlands	1	56.1	59.7	26.8	31.3	36	32	8.9	9.0	149.2	172.3
	2	39.3	46.1	25.0	30.0	128	107	15.5	17.9	233.9	317.7
	3	34.2	35.5	24.0	28.2	22	22	2.0	2.2	28.9	35.3
	4	20.0	24.1	19.6	24.1	7	3	0.2	0.1	2.5	1.8
	5	32.0		23.0	-	1	-	0.1	-	1.1	-
	Total	41.9	46.9	25.5	30.2	194	169	26.7	29.2	415.7	527.3

[a]Social position was determined following Kraft (1884). Social position indicates the vitality and competitive intensity of trees. The Kraft social classes describe the current position of a tree relative to height in a mostly even-aged forest with a homogenous structure (Chirici et al., 2011). The Kraft classification is as follows: 1 = predominant, 2 = dominant, 3 = low co-dominant, 4 = dominated, 5 = suppressed, entirely overtopped trees (Pretsch 2009).

Bibliography

Kraft G (1884) Zur Lehre von den Durch Forstungen. Schlagstellungen und Lichtungshieben, Hanover.

Chirici G, Winter S, McRoberts RE (2011). Managing Forest Ecosystems. National Forest Inventories: Contributions to Forest Biodiversity Assessments. Springer Verlag, 224 pp.

Pretsch H (2009) Forest Dynamics Growth & Yield. Springer-Verlag, 664 pp.
