# Supplementary material for: Within-Population Genetic Structure in Beech (Fagus sylvatica L.) Stands Characterized by Different Disturbance Histories: Does Forest Management Simplify Population Substructure?
Source: PLoS One. 2013 Sep 5;8(9):e73391. doi: 10.1371/journal.pone.0073391 (PMC3764177; doi:10.1371/journal.pone.0073391)
Supplement: Table S2 — Heterogeneity tests of SGS between disturbed and undisturbed plots using GenAlEx (Peakall & Smouse [30] ). The reported values represent the degree of differentiation of SGS between two plots from the same site in each distance class (t2) and total ω. (PDF) [file pone.0073391.s003.pdf]

Table S2 Heterogeneity tests of SGS between disturbed and undisturbed plots using GenAlEx (Peakall & Smouse [30]). The reported values represent the degree of differentiation of SGS between two plots from the same site in each distance class (t^2) and total ω

	Distance class (t^2)									
Pairs of plots	0-20 m		20-40 m	40-60 m	60-80 m		80-100 m		Total (ω)	
Gh vs. Gl	4.22	*	0.94	0.37	1.42		1.72		8.07	
NLh vs. NLl	0		2.91	1.41	0.75		0.34		5.43	
Ah vs. Al	0.98		3.6	0.62	0.05		1.82		6.74	
Fh vs. Fl	22.76	***	2.14	0.15	7.58	**	7.62	**	21.84	***
Ih vs. Il	22.21	***	0.04	0.01	2.18		NA		11.43	**

*P<0.05, **P<0.01, ***P<0.001
