# Supplementary material for: Within-Population Genetic Structure in Beech (Fagus sylvatica L.) Stands Characterized by Different Disturbance Histories: Does Forest Management Simplify Population Substructure?
Source: PLoS One. 2013 Sep 5;8(9):e73391. doi: 10.1371/journal.pone.0073391 (PMC3764177; doi:10.1371/journal.pone.0073391)
Supplement: Text S1 — Assessment of the potential impact of null alleles on spatial correlation analyses in the studied Fagus sylvatica stands. (PDF) [file pone.0073391.s004.pdf]

Text S1 – Assessment of the potential impact of null alleles on spatial correlation analyses in the studied *Fagus sylvatica* stands

To assess the potential impact of null alleles on results from spatial autocorrelation analyses we modified the French datasets randomly introducing null alleles according to the frequencies resulting from progeny tests by Piotti et al. (2012).

Null alleles were introduced by transforming: 1) homozygote individuals in heterozygotes with a null allele, and 2) non-amplifying genotypes (i.e. no amplification products) in homozygotes for the null alleles (see Bacles & Ennos 2008, Piotti et al. 2012). The procedure was applied to French datasets because null alleles frequencies were estimated on progenies from those plots for the 4 microsatellites used in the present work (Piotti et al. 2012). Once the datasets were modified, spatial autocorrelation was run in GenAlEx (Peakall & Smouse 2012). The procedure was independently replicated 5 times. Results showed that both the general SGS pattern and single distance-class values obtained from modified datasets are very close to what found using the original dataset, thus showing a non significant effect of the possible presence of null alleles on SGS (Figure TS1).

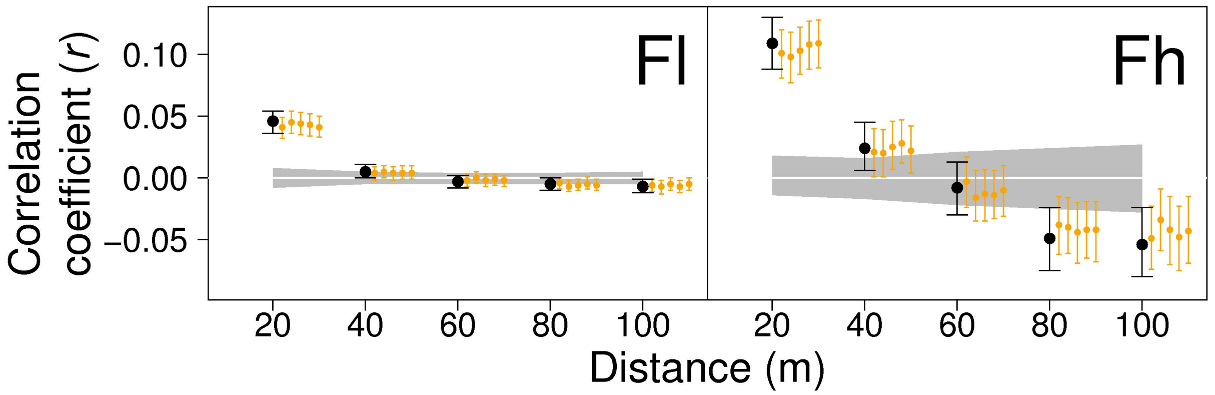

Figure TS1 – Comparison of spatial autocorrelation results obtained from the original (black dots) and modified datasets in which null alleles were introduced (orange dots). Black and orange lines around mean *r* values represent 95% confidence intervals generated by bootstrapping (1000 times) pair-wise comparisons within each distance class.

Bibliography

Bacles CFE, Ennos RA (2008) Paternity analysis of pollen-mediated gene flow for *Fraxinus excelsior* L. in a chronically fragmented landscape. Heredity 101: 368–380.

Peakall R, Smouse PE (2012) GenAlEx 6.5: genetic analysis in Excel. Population genetic software for teaching and research-an update. Bioinformatics 28: 2537-2539.

Piotti A, Leonardi S, Buiteveld J, Geburek T, Gerber S, et al. (2012) Comparison of pollen gene flow among four European beech (*Fagus sylvatica* L.) populations characterized by different management regimes. Heredity 108: 322-331.
